# Correction to “Trametinib Overcomes *KRAS* ‐G12V–Induced Osimertinib Resistance in a Leptomeningeal Carcinomatosis Model of *EGFR* ‐Mutant Lung Cancer”

**DOI:** 10.1111/cas.70278

**Published:** 2025-11-24

K. Fukuda, S. Otani, S. Takeuchi, et al., “Trametinib Overcomes *KRAS*‐G12V–Induced Osimertinib Resistance in a Leptomeningeal Carcinomatosis Model of *EGFR*‐Mutant Lung Cancer,” Cancer Science 112 (2021): 3784–3795. https://doi.org/10.1111/cas.15035.

In the above article, Figure 6B is incorrect. In Figure 6B, the image for “Control, Day 7” was inadvertently duplicated from “Osimertinib, Day 7” during figure assembly. The corrected panel is shown below. The color scale and radiance range are identical to the published figure (1 × 10^6^ – 2 × 10^8^ p/sec/cm^2^/sr). This error is limited to the representative image and does not affect the quantitative data in Figure 6A, the text, or the conclusions of the article. We apologize for the oversight.

The correct image is shown below:

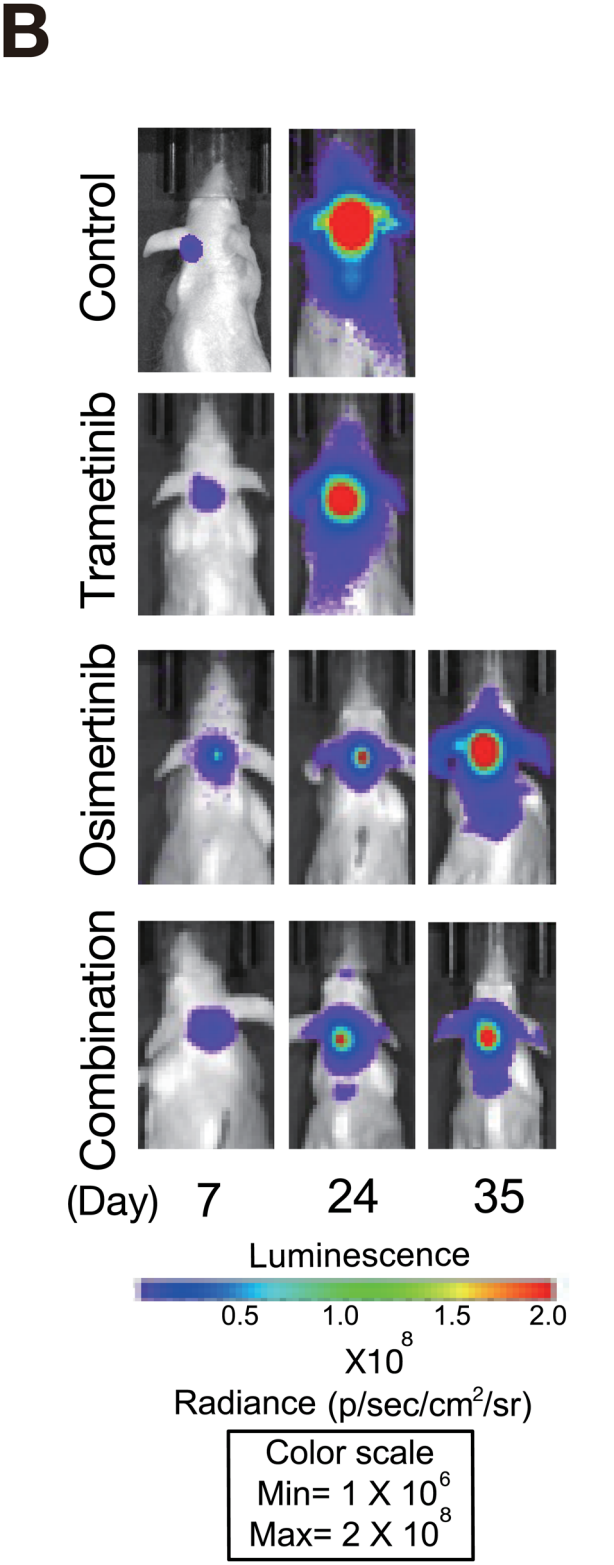

We apologize for this error.

